# Immunogenicity analysis of conserved fragments in *Plasmodium ovale* species merozoite surface protein 4

**DOI:** 10.1186/s12936-020-03207-7

**Published:** 2020-03-30

**Authors:** Juliette Uwase, Ruilin Chu, Kokouvi Kassegne, Yao Lei, Feihu Shen, Haitian Fu, Yifan Sun, Yinghua Xuan, Jun Cao, Yang Cheng

**Affiliations:** 1 Laboratory of Pathogen Infection and Immunity, Department of Public Health and Preventive Medicine, Wuxi School of Medicine, Jiangnan University, Wuxi, 214122 Jiangsu People’s Republic of China; 2 Key Laboratory of National Health and Family Planning Commision on Parasitic Disease Control and Prevention, Jiangsu Provincial Key Laboratory on Parasite and Vector Control Technology, Jiangsu Institute of Parasite Diseases, Wuxi, 214064 Jiangsu People’s Republic of China

**Keywords:** *Plasmodium ovale* species, MSP4, Conservation, Immunogenicity, Cross-reactivity, Avidity index

## Abstract

**Background:**

There is an urgent need for an effective vaccine to control and eradicate malaria, one of the most serious global infectious diseases. *Plasmodium* merozoite surface protein 4 (MSP4) has been listed as a blood-stage subunit vaccine candidate for malaria. Infection with *Plasmodium ovale* species including *P. ovale wallikeri* and *P. ovale curtisi*, is also a source of malaria burden in tropical regions where it is sometimes mixed with other *Plasmodium* species. However, little is known about *P. ovale* MSP4.

**Methods:**

The *msp4* gene was amplified through polymerase chain reaction using genomic DNA extracted from blood samples of 46 patients infected with *P. ovale* spp. and amplified products were sequenced. Open reading frames predicted as immunogenic peptides consisting of 119 and 97 amino acids of *P. ovale curtisi* MSP4 (PocMSP4) and *P. ovale wallikeri* MSP4 (PowMSP4), respectively, were selected for protein expression. Recombinant proteins (rPoMSP4) were expressed in *Escherichia coli*, purified, analysed, and immunized in BALB/c mice. The specificity of anti-MSP4-immunoglobulin (Ig) G antibodies was evaluated by Western blot and enzyme-linked immunosorbent assays, and cellular immune responses were analysed via lymphocyte proliferation assays.

**Results:**

Full peptide sequences of PocMSP4 and PowMSP4 were completely conserved in all clinical isolates, except in the epidermal growth factor-like domain at the carboxyl terminus where only one mutation was observed in one *P. o. wallikeri* isolate. Further, truncated PoMSP4 segments were successfully expressed and purified as ~ 32 kDa proteins. Importantly, high antibody responses with end-point titres ranging from 1:10,000 to 1:2,560,000 in all immunized mouse groups were observed, with high IgG avidity to PocMSP4 (80.5%) and PowMSP4 (92.3%). Furthermore, rPocMSP4 and rPowMSP4 cross-reacted with anti-PowMSP4-specific or anti-PocMSP4-specific antibodies. Additionally, anti-PoMSP4 IgG antibodies showed broad immuno-specificity in reacting against rPoMSP1 and rPoAMA1. Lastly, PocMSP4- and PowMSP4-immunized mice induced cellular immune responses with PocMSP4 (36%) and PowMSP4 cells (15.8%) during splenocyte proliferation assays.

**Conclusion:**

Findings from this study suggest conservation in PoMSP4 protein sequences and high immunogenicity was observed in rPoMSP4. Furthermore, induction of immune responses in PocMSP4- and PowMSP4-immunized mice informed that both humoral and cellular immune responses play crucial roles for PoMSP4 in protection.

## Background

Eradication of malaria is still among the major priorities in the malaria research agenda as the disease continues to kill thousands of peoples worldwide [1]. In 2017, the World Health Organization (WHO) estimated 219 million cases of malaria and 435,000 deaths, a figure that was assumed too high [2]. Effective vaccine development was emergently required to enhance existing malaria control measures because of the moderate spread of drug and insecticide resistance. Recently, some African countries applied one vaccine RTS, S/AS01; however, this vaccine only targets *Plasmodium falciparum* [3–5] and might probably be inadequate in areas where a remarkable proportion of patients suffers from *Plasmodium vivax* or mixed infections. Moreover, three other parasitic species, namely, *Plasmodium ovale*, *Plasmodium malariae,* and *Plasmodium knowlesi*, can cause malaria infection. *Plasmodium ovale* can cause malaria infection in humans but has lower incidence compared with *P. falciparum* and *P. vivax* [6, 7]. Some cases of *P. ovale* infection can occur in endemic areas of malaria where other species co-exist [8, 9]; therefore, such evidence should be considered in malaria control strategies. *Plasmodium ovale* has been separated into two distinct species (*P. ovale curtisi* and *P. ovale wallikeri*) in 2010 [10–12]. Similar to other malaria parasites of primates, *Anopheles* species are able to transmit *P. ovale;* the parasites then invade reticulocytes and begin the erythrocytic cycle that might last approximately 49 h [7].

Hosts respond to malaria parasites by generating antibodies against parasite-derived antigens, and naturally acquired immunity is developed after repeated exposure to infections [13]. Notably, antibodies against merozoite antigens play a significant role in conferring immunity against malaria [14, 15]. Asexual-stage antigens located on apical organelles or on the surfaces of merozoites offer considerable potential as components of vaccines against malaria. Immune responses induced by such vaccines can block the invasion of host erythrocytes through merozoites [16]. Thus, malaria antigens recognized as candidates for vaccine development are generally grouped as pre-erythrocytic, erythrocytic, and transmission-blocking antigens. Antigens in the asexual stages of malaria parasites represent potential targets for malaria vaccines. Blood-stage vaccines point to target the subsequent disease-causing stage of the *Plasmodium* life cycle and may provide protection against disease severity, reducing blood stage asexual parasitaemia and transmission [17]. Merozoite surface protein 1 (MSP1) and apical membrane antigen (AMA1) are leading blood-stage malaria antigens and considered important vaccine candidates [15], especially due to their association with protection in pre-clinical studies of mice and non-human primates [18–20]. Protection is associated with the induction of high-titre antibodies. Several studies have investigated immune cross-reactivity of antigens in erythrocytic asexual blood stages. For example, immune sera and monoclonal antibodies against AMA1 manifested only limited cross-reactivity between *P. falciparum* and *P. vivax* [21]. Similar studies using sera from people infected with *P. falciparum* and *P. vivax* showed cross-reactivity of merozoite surface protein 5 (MSP5)-specific antibodies [22]. Evidence of immune response has been reported for the asexual erythrocytic stages of *P. falciparum* and *P. vivax* antigens, but there is limited information on antigens of *P. ovale* species.

Merozoite surface protein 4 (MSP4) is a glycosylphosphatidylinositol-anchored protein that contains an epidermal growth factor (EGF)-like domain at the carboxyl terminus [23–25]. MSP4 protein has been shown immunogenic in laboratory animals [25] and crucial for parasite survival [26]. Murine models of malaria showed that this protein can induce protective immunity against lethal challenge and protect against heterologous challenge by a different species of murine malaria [27, 28]. Immunization with recombinant *Plasmodium yoelii* MSP4/5 in mice, a homolog of MSP4 and its related antigen MSP5, has induced protective immune responses against lethal parasite challenge with *P. yoelii* [27, 28]; protection is enhanced when MSP4/5 is immunized in combination with *P. yoelii* MSP1 [29]. This finding suggested that MSP4 is a potential malaria vaccine candidate, especially in combination with other antigens. MSP4 immunogenicity has been associated with protection in natural infections with *P. falciparum* [30–32]. In addition, MSP4 shows a high degree of conservation among *P. falciparum* isolates [16, 33, 34], supporting its potential consideration as a subunit component for malaria vaccine formulations. However, information on *P. ovale* MSP4 anti-malarial properties of immunity is scant.

In this study, MSP4 sequences were analysed in clinical isolates of *P. o. curtisi* and *P. o. wallikeri* from infected subjects to assess conservation and immunogenicity of *P. ovale* spp. MSP4. Furthermore, recombinant PoMSP1 and PoAMA1 antigens were tested against anti-PoMSP4 immunoglobulin G (IgG) antibodies to evaluate the specificity of PoMSP4 antigens.

## Methods

### Malaria samples

*P.o. curtisi* and *P. o. wallikeri* infected blood samples were obtained from local hospitals in Jiangsu Province (China) between 2012 and 2016 from febrile patients who had returned from work in malaria endemic areas of sub-Saharan Africa [35]. Identification of the isolates was confirmed by polymerase chain reaction (PCR) analysis, and parasite species were distinguished using real-time TaqMan PCR [36].

### PCR amplification and sequencing of *pomsp4* genes

Genomic DNA extracted from *P. ovale*-infected individual blood samples was previously preserved in our laboratory. A total of 46 *P. ovale* spp. genomes (*P. o. curtisi*, n = 23 and *P. o. wallikeri*, n = 23) were randomly selected for amplification. Information on the imported *P. ovale* spp. specimens is given in Additional file 1: Table S1. Full nucleotide sequences of *pocmsp4* and *powmsp4* were amplified via PCR using primers designed as follows: *pocmsp4* forward (5′-ATG AGG GTA CTC CAA TTT TTA TTA C-3′), *pocmsp4* reverse (5′-TTA ATT TAT TGA CGC TAA AAT G-3′), *powmsp4* forward (5′-ATG AGG GTA CTC CAA TTT TTA TTA C-3′), and *powmsp4* reverse (5′-TTA ATT TAT TGA CGC TAA AAT G-3′). *Pocmsp4* (*Plasmodium* Genomics Resource database, PocGH01_04023000) and *powmsp4* (National Centre for Biotechnology Information GenBank database, accession number: LT594508.1) were used as reference gene sequences. Reactions were carried out in a volume of 20 μL, including 1 μL of genomic DNA, 0.8 μL of each primer (10 µM), 7.4 μL of double-distilled water, 0.5 units of DNA polymerase, and 2 mM deoxynucleoside triphosphate within 10 μL of premix (2× Phanta^®^ Max Master Mix, Vazyme). PCR amplification was performed in a Mastercycler (Eppendorf) as follows: denaturation at 95 °C for 3 min; 35 cycles of 95 °C for 15 s, 51 °C for 30 s, and 72 °C for 30 s; and final extension at 72 °C for 5 min. PCR products were analysed in 1% agarose gel electrophoresis, visualized under an ultraviolet transilluminator (Bio-Rad ChemiDoc MP), and sequenced by Genewiz.

### Protein bank

The N-terminal of PoMSP1 [*pocmsp1* (GenBank: KC137343) and *powmsp1* (GeneBank: KC137341)] and full length of PoAMA1 [*pocama1* (PlasmoDB: PocGH01_09039800) and *powama1* (GenBank: SBT36045.1)] merozoite surface proteins, which were previously expressed and preserved in our laboratory, were used for specificity tests of PoMSP4 protein-raised antibodies.

### Construction of recombinant *pomsp4* clones

PoMSP4-predicted open reading frames (ORFs) without the EGF-like domain consisting of 1–119 (PocMSP4) and 1–97 (PowMSP4) amino acids, for which a *P. falciparum* ortholog has been reported as highly immunogenic [30, 31, 37], were selected. Genomic DNA from *P. ovale* isolates was used as template for PCR amplification of *pomsp4* ORFs. Primers were as follows: *pocmsp4* forward (5′-ATG AGG GTA CTC CAA TTT TTA TTA C-3′), *pocmsp4* reverse (5′-AGG CGA TGC TAT CGG TTT TG-3′), *powmsp4* forward (5′-ATG AGG GTA CTC CAA TTT TTA TTA C-3′), and *powmsp4* reverse (5′-TGC TAT ACC TAG GAC ATT TTT ACC C-3′). The reaction was performed in a 20 µL volume as described above on a Mastercycler (Eppendorf) with the following temperature profile: initial denaturation at 95 °C for 3 min; 35 cycles of 95 °C for 15 s, 56 °C for 30 s, and 72 °C for 30 s; and a final extension at 72 °C for 5 min. PCR products were also analysed as described above.

The amplified fragments were cloned into the pUC57 vector, sequenced by Genewiz on an ABI 3730xl DNA analyzer (Thermo Fisher Scientific) using universal primers (M13F: 5′-TGT AAA ACG ACG GCC AGT-3′, M13R: 5′-CAG GAA ACA GCT ATG AC-3′), and subcloned into pET32a expression plasmid vector (YouLong Biotech). Recombinant plasmids were transformed into expression host *Escherichia coli* strain BL21 (DE3) pLysS and sequenced using universal primers T7 through Genewiz.

### Protein expression and purification

*Escherichia coli* BL21 (DE3) pLysS cells containing recombinant plasmid pET32a^*pomsp4*^ were cultured in Luria–Bertani (supplemented with 50 mg/mL ampicillin) at 37 °C with shaking until optical density (OD) of 600 nm reached 0.6–0.8. The culture was induced with 0.5 mM isopropyl β-d-1 thiogalactopyranoside and allowed to grow for another 3 h at 37 °C. Cells were harvested through centrifugation at 4000×*g* for 30 min. Protein purification was performed by YouLong Biotech using the following technique. Thawed cells were suspended in purification buffer [50 mM Tris–HCl (pH 8.0), 300 mM NaCl, and 10 mM imidazole] and lysed by sonication. The insoluble fraction was separated by centrifugation at 15,000×*g* for 15 min at 4 °C. The soluble fraction was applied to a column containing 1.0 mL of Ni-nitrilotriacetic acid-agarose (Qiagen) and then washed with 10 mL of purification buffer containing 20 mM imidazole. Recombinant proteins were eluted from the column with purification buffer containing 250 mM imidazole and then exchanged into Tris–HCl storage buffer [50 mM Tris–HCl (pH 8.5), 100 mM NaCl, 1 mM DTT, 0.1 mM phenylmethylsulfonyl fluoride, and 10% (v/v) glycerol] using a 30 kDa ultrafiltration tube (Millipore). Proteins were stored at − 80 °C until use.

### Analyses of protein

The concentration of recombinant proteins (rPoMSP4) was determined through the Bradford method using bovine serum albumin (BSA) as standard (Bradford protein assay kit, Solarbio). Purified proteins were analysed by 12% sodium dodecyl sulfate–polyacrylamide gel electrophoresis (SDS-PAGE) and Coomassie brilliant blue staining (Beyotime Biotech) to assess the expression level and immunoreactivity. The separated proteins from SDS-PAGE were electrophorectically transferred onto a polyvinylidene difluoride (PVDF) membrane (Immobilon) and blocked overnight in Tris-buffered saline with 0.1% Tween-20 (TBST) containing 5% skimmed milk at 4 °C. The membranes were probed with anti-His antibody (ABclonal) at 1:5000 dilution along with primary antibody dilution buffer (Meilunbio) overnight at 4 °C. Membranes were washed three times with 0.1% TBST and treated with horseradish peroxidase (HRP)-conjugated goat anti-mouse IgG (Cowin Biotech) at 1:5000 dilution for 90 min. Finally, the membranes were analysed with a ChemiDoc MP imaging system (Bio-Rad).

### Antibody raising and immunodetection

Six- to eight-week-old female BALB/c mice were used for immunizations as follows. Mice were grouped into the rPoMSP4-immunized (n = 5 per group) and negative control groups (n = 3 per group). Each mouse was intraperitoneally injected with 50 µg of rPocMSP4, rPowMSP4, or PBS, all of which were diluted in PBS with complete Freund’s adjuvant (Sigma). An equal volume of antigen with incomplete Freund’s adjuvant (Sigma) was used for subsequent boosters, which were administered on days 21 and 42 post-immunization intraperitoneally. The control group was administered an equal amount of PBS and adjuvant. Mouse blood samples were collected from the tip of the tail on days 0, 7, 14, 28, 35, and 49. Sera were obtained via centrifugation for 20 min at 2000 × rpm and stored at − 80 °C.

Purified rPoMSP4 was tested against sera from rPocMSP4- or rPowMSP4-infected mice to assess anti-PoMSP4 IgG antibodies through Western blot analysis. During the assays, PVDF membranes were incubated with antisera (1:2000 dilutions) from the rPoMSP4-immunized group or negative control group, followed by HRP-conjugated goat anti-mouse IgG (Cowin Biotech) at 1:5000 dilution.

### Specificity of anti-PoMSP4 antibodies

Levels of IgG antibodies targeting PoMSP4 in mouse sera were detected via enzyme-linked immunosorbent assays (ELISA). In brief, 96-well ELISA plates were coated with 50 ng of rPoMSP4 antigen dissolved in coating buffer solution (15 mM sodium carbonate and 35 mM sodium bicarbonate in distilled water) overnight at 4 °C. After three times wash with PBS containing 0.1% of Tween-20 (PBST), the plates were blocked with 1% BSA in PBS and incubated at room temperature for 2 h. Thereafter, individual mouse sera (100 µL) diluted at different dilutions were added on the plate and incubated at room temperature for 2 h. The plates were washed again three times with PBST and HRP-conjugated goat anti-mouse IgG antibodies (Southern Biotech) at 1:5000 dilution and incubated for 1 h 30 min at room temperature. The plates were finally washed three times with PBST and incubated with 3,3′,5,5′-tetramethylbenzidine (Invitrogen) substrate for a few minutes in the dark, and 2 M H_2_SO_4_ was added to stop the reaction. The absorbance at OD of 450 nm was measured using a microplate reader (Synergy, BioTeK). Furthermore, anti-PoMSP4 IgG antibodies were tested against rPoMSP1 and rPoAMA1 antigens via ELISA to test for the specificity of antibodies.

### Lymphocyte proliferation assays

Lymphocyte proliferation was measured using a cell counting kit-8 (CCK-8, Beyotime Biotech). A certain amount of 5 × 10^5^ cells/well of PoMSP4- and PBS-immunized cells was treated with 10 µL of PocMSP4 (5 µg/mL), 10 µL of PowMSP4 (5 µg/mL), or 10 µL of concanavalin A (Con A, 2 μg/mL), as positive control, in 96-well flat-bottom microtitre plates and then incubated for 72 h at 37 °C with 5% CO_2_. Thereafter, 10 µL of CCK-8 was added to each well, and the plates were incubated for 2 h at 37 °C and measured at 450 nm using a microplate reader.

### Sequence alignment and analysis of data

Full nucleotide sequences of *pomsp4* genes from all clinical isolates were translated to the deduced amino acid sequences using the MegAlign module of Lasergene 7 software package (DNAstar) and then aligned with reference sequences to assess conservation within PoMSP4. Amino acid sequences of the segments of PocMSP4 and PowMSP4 isolates were aligned with those of the PocGH01_04023000 and LT594508.1 reference strains, respectively. Sequence alignment for all *P. ovale* isolates was performed using MEGA v.7.0 software.

Statistical analysis and graphing were conducted using GraphPad Prism software version 5.0 (Graph Pad software, Inc.). SPSS v.16.0 was performed to analyse cross-reaction and antibody responses. Student’s t-test with probability (*P*) value of < 0.05 indicated a significant difference.

## Results

### Analysis of amplified *pomsp4* genes revealed conservation of amino acids in isolate sequences

**Fig. 1:**
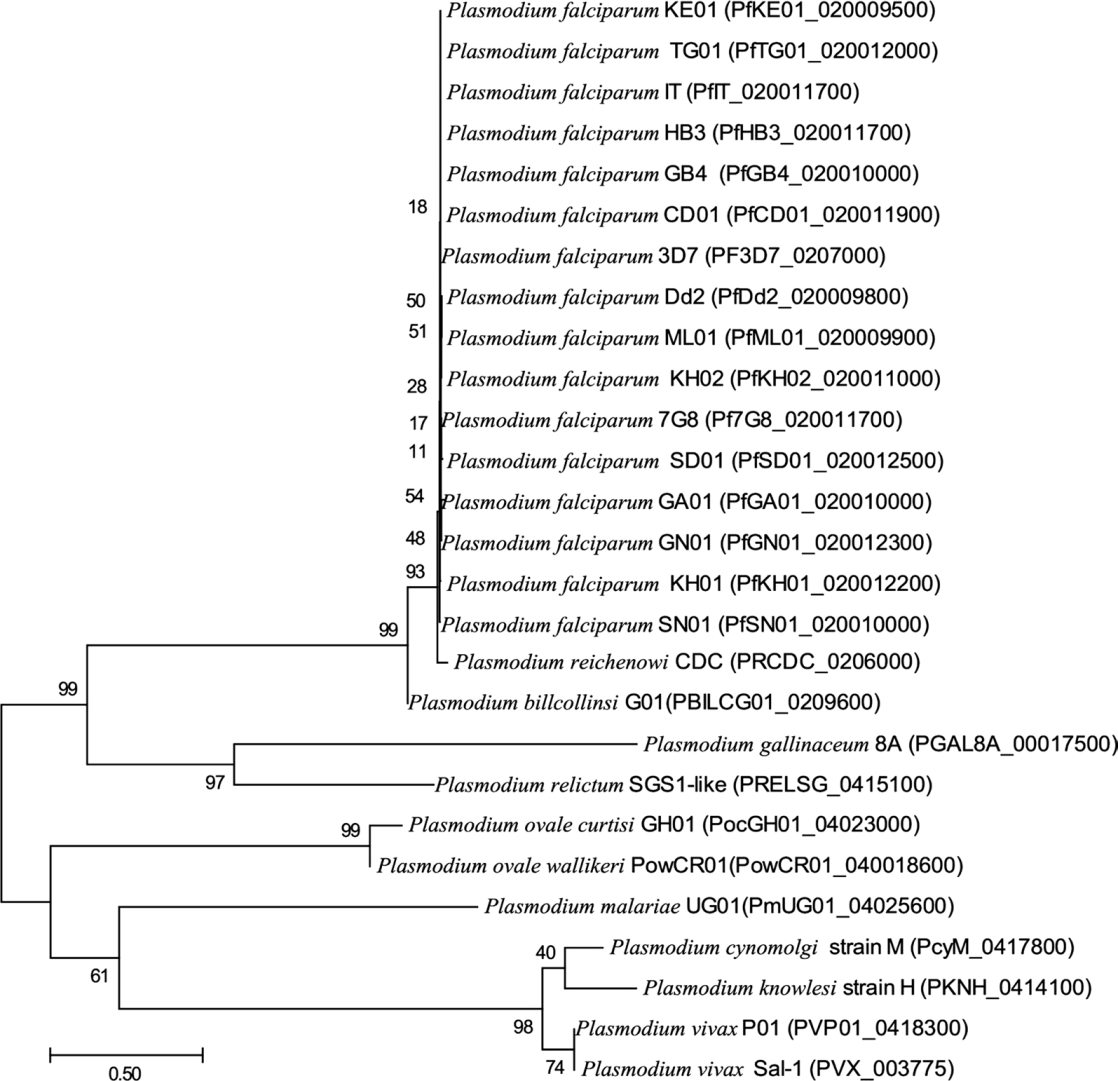
Phylogenetic analysis of *msp4* gene sequences within orthologues in other *Plasmodium* species using the neighbour-joining method. The analysis of *pocmsp4* and *powmsp4* revealed 99% similarity

The full length of *pomsp4* genes was successfully amplified from the genomic DNA of 23 *P. o. curtisi* and 23 *P. o. wallikeri* infected individuals. A phylogenetic tree was constructed through the neighbour-joining method based on human, non-human primate, murine, and avian malaria species to infer genetic relationships of *pocmsp4* and *powmsp4*. Phylogenetic analysis of gene sequences of both *pocmsp4* and *powmsp4* revealed 99% similarity (Fig. 1). The *msp4* gene ID number of the *plasmodium* species included in the analyses is provided in Additional file 2: Table S2 The alignments of PoMSP4 amino acid sequences from both subspecies showed that no amino acid mutation occurred among all isolate samples from 17 different countries in Sub-Saharan Africa (Additional file 3: Figure S1). This suggests complete conservation of PoMSP4, which is consistent with previous findings, especially in *P. falciparum* orthologs [16, 33, 34]. Only one mutation was observed in the EGF-like domain at the carboxyl terminus in one *P. o. wallikeri* isolate (from Uganda); however, this single mutation could not affect the antigenicity of MSP4 proteins because EGF domains have shown relatively poor immunogenicity [30, 31, 37].

### Expression, purification, and analysis of recombinant PoMSP4 proteins

**Fig. 2:**
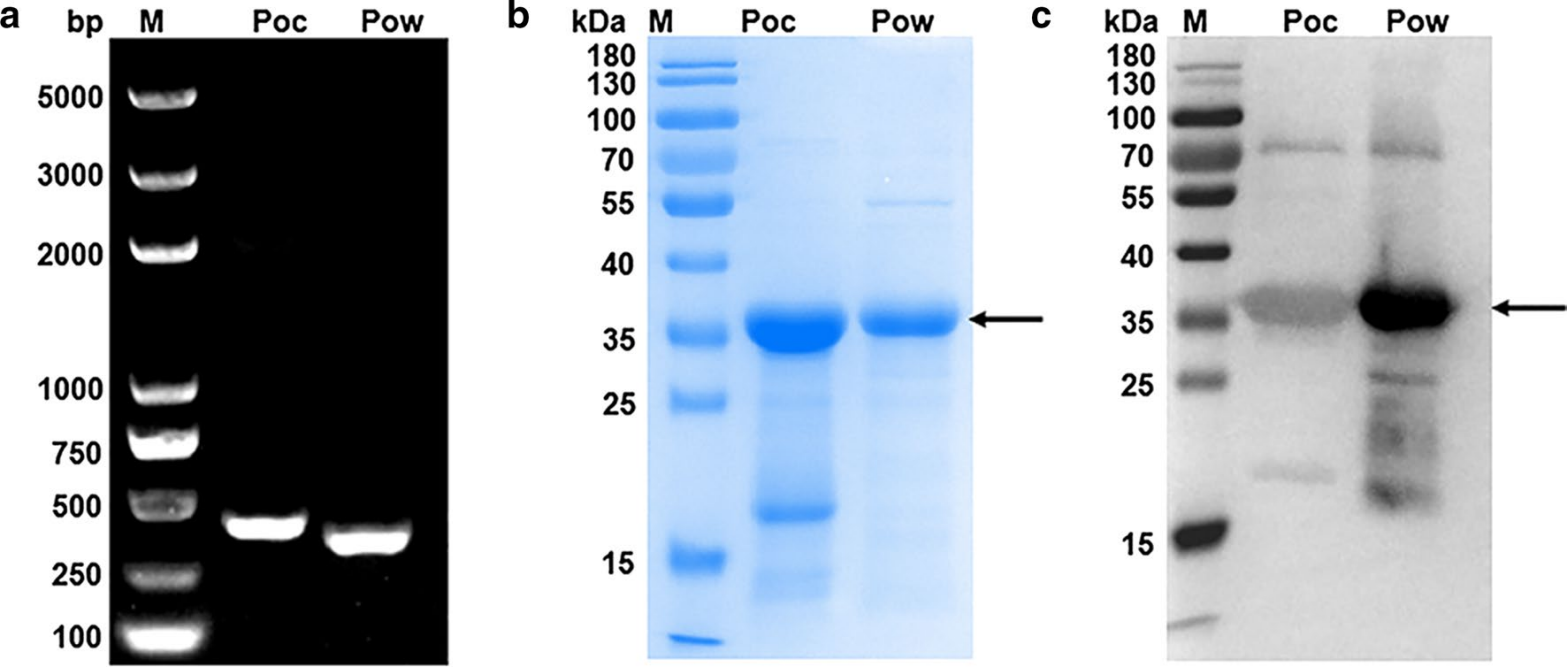
**a** Target genes of 46 *P. ovale* spp. isolates were amplified via PCR, and accurate sizes of 360 and 294 bp were obtained for *pocmsp4* and *powmsp4*, respectively. **b** Purified PoMSP4 proteins analysed through SDS-PAGE with Coomassie blue staining. Analysis of rPoMSP4 showed that *pocmsp4* and *powmsp4* had a molecular weight of approximately 32 kDa. **c** Western blot analysis of rPoMSP4 using anti-His antibody indicated the specific bands. Molecular weight markers are indicated in kilodaltons (kDa)

The selected *pomsp4* fragments were successfully amplified in isolates through PCR, generating single PCR products with the expected size of 360 bp for *pocmsp4* and 294 bp for *powmsp4*. Direct sequencing of purified PCR fragments showed no superimposed signal on the electropherograms for *pomsp4* (Fig. 2a).

The molecular weight of expressed PocMSP4 and PowMSP4 ORFs was estimated to be approximately 32 kDa, which was consistent with that obtained in purified proteins as shown in SDS-PAGE analysis with Coomassie blue staining (Fig. 2b). Immunoblotting analysis that used anti-His tag antibody confirmed that rPoMSP4 was expressed (Fig. 2c).

### Mice-derived antibodies against PoMSP4 recognized the recombinant proteins

**Fig. 3:**
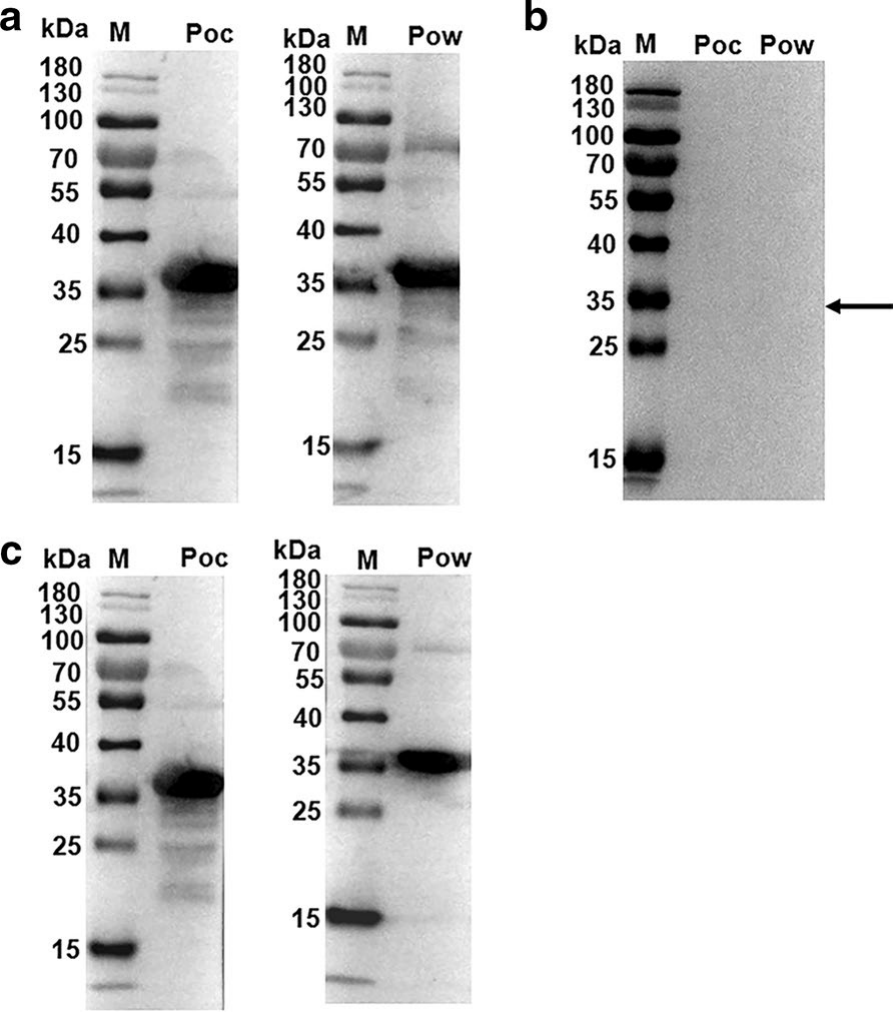
Reactivity of serum from mice immunized with rPoMSP4. **a** Western blot analysis for the detection of rPoMSP4 proteins using sera from mice immunized with rPoMSP4 proteins. **b** Sera from mice immunized with PBS and adjuvant as negative control. **c** Cross-reactivity of rPoMSP4 with sera of mice immunized with recombinant PoMSP4 was verified by Western blot. Molecular weight markers are indicated in kilodaltons (kDa)

An immunoblot specific to 32 kDa bands corresponding to the sizes of the two rPoMSP4 proteins was elaborated to determine whether mice anti-rPoMSP4 antibodies can identify rPoMSP4 (Fig. 3a). The antibody responses of the sera from immunized mice were potent against rPoMSP4, suggesting that PoMSP4 could induce immune responses and was immunogenic in mice. No reactivity was noted in the normal and PBS-immunized mice negative controls (Fig. 3b). Furthermore, cross-reactivity was tested using anti-rPoMSP4-immune mouse sera with each of the rPoMSP4 proteins (Fig. 3c). These results showed that mice anti-rPocMSP4 and anti-rPowMSP4 antibodies could recognize rPowMSP4 and rPocMSP4 antigens, respectively.

### Immune responses against rPoMSP4 in mice

**Fig. 4:**
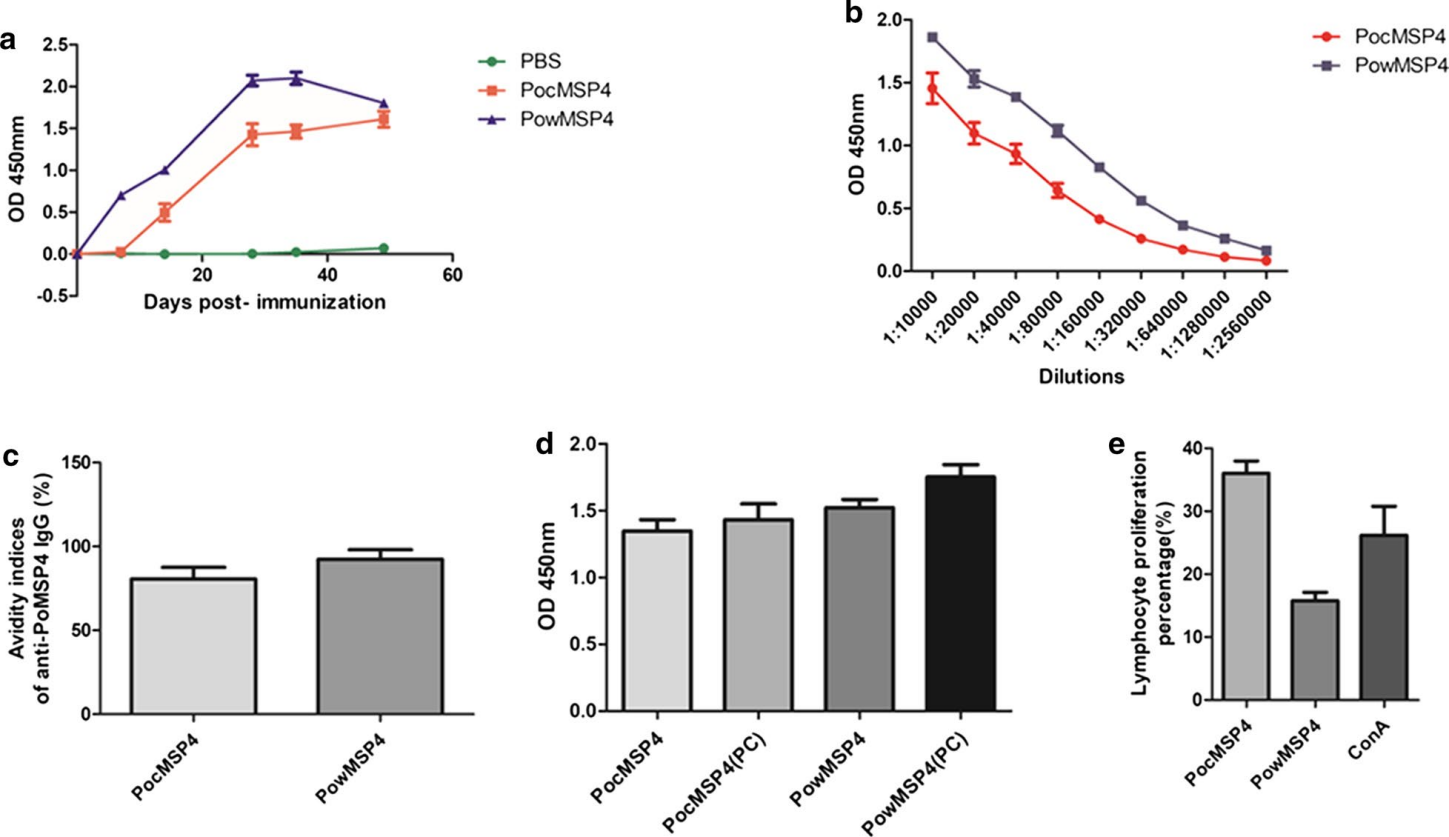
Immune response in mice immunized with rPoMSP4. **a** IgG was detected at day 7 post-immunization, and the levels continued to rise throughout the whole immunization period. **b** Immunized mice sera were diluted from 1:10,000 to 1:2,560,000. Data are presented as the geometric mean OD obtained at different concentrations. Numbers on the X-axis indicate the dilutions tested. Antigen specificity was confirmed using pre-immune serum samples as control. **c** Antibody avidity of rPoMSP4 was verified through ELISA. Antibody avidity was higher in PowMSP4 (mean 92.3) than in PocMSP4 (mean 80.5). **d** Cross-reaction of PocMSP4 with sera from mice immunized with rPowMSP4 proteins and cross-reaction of PowMSP4 with sera from mice immunized with rPocMSP4 (PC, positive control). **e** Splenocytes of immunized BALB/c mice were stimulated with 5 µg/mL of each recombinant protein used in immunization. The cells were stimulated for 72 h. Data were measured at 450 nm and transformed in percentages of PocMSP4 (36%) and PowMSP4 (15.8%)

Antibody responses from all groups of mice were measured through ELISA using the same recombinant proteins constructed as the solid phase coating antigen. ELISA results showed that antibodies against PoMSP4 in mice could be detected 1 week after the primary booster. A response was detected at day 7 post-immunization and continued to rise 00 (Fig. 4a). Mean serum antibody titres were evaluated through ELISA at 49 days after the first immunization. Similarly, PocMSP4 and PowMSP4 induced a high antibody response with end-point titres ranging from 1:10,000 to 1:2,560,000 (Fig. 4b). Anti-rPocMSP4 and anti-rPowMSP4 antibodies in all immunized mouse groups exhibited high avidity indexes (AIs), and the average AIs of anti-rPocMSP4 and anti-rPowMSP4 IgGs were 80.5% and 92.3%, respectively (Fig. 4c).

### Cross-reactivity of rPoMSP4 proteins with anti-rPoMSP4 antibodies

ELISA was used to investigate whether the antibodies from rPocMSP4-immunized mice can combine with PowMSP4 protein and whether the antibody of rPowMSP4-immunized mice can combine with PocMSP4 protein. The results showed that mouse anti-rPocMSP4 or anti-rPowMSP4 antibodies could recognize rPocMSP4 and rPowMSP4, with no significant difference (*p *= 0.5758; Fig. 4d), suggesting the high specificity of both anti-PocMSP4 and anti-PowMSP4 antibodies.

### Lymphocyte proliferation assays

PocMSP4 and PowMSP4 protein antigens were able to induce high antibody levels in mice. Therefore, spleen lymphocyte proliferation assays were used to test whether these antigens can induce cellular immune responses. The proliferation assay was performed to assess the influence of the splenocyte proliferative response to rPoMSP4 proteins in vitro under rPocMSP4, rPowMSP4, and ConA (positive control) stimulations. Results showed that rPoMSP4-induced cell proliferation was at 36% for PocMSP4 and 15.8% for rPowMSP4 (Fig. 4e).

### Specificity of PoMSP4-derived antisera

**Fig. 5:**
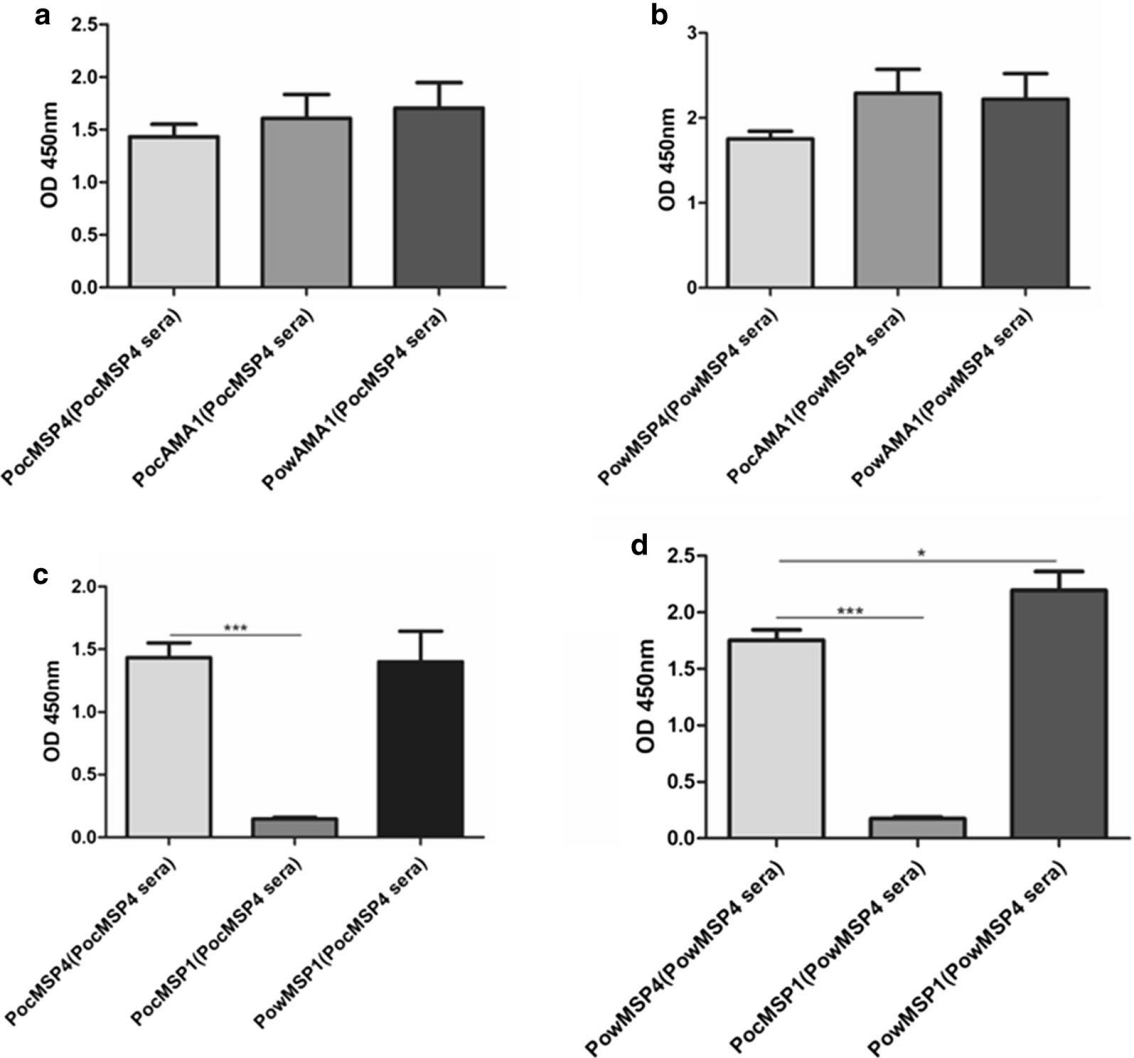
Specificity and reactivity of the sera of rPoMSP4-immunized mice using PoAMA1 and PoMSP1 proteins. The reactivity of sera from mice immunized with rPoMSP4 was evaluated using rPoAMA1 and rPoMSP1 proteins. **a**, **b** Cross-reaction of rPoAMA1 proteins with antisera from mice immunized with rPoMSP4. No significant differences were observed between PocMSP4, PocAMA1, or PowAMA1 and the anti-PocMSP4 antisera and between PowMSP4, PocAMA1, or PowAMA1 and the anti-PowMSP4 antisera (*p *> 0.05). **c**, **d** Cross-reaction of rPoMSP1 proteins with antisera from mice immunized with rPoMSP4. Significant differences between groups are indicated on the graph. No significant difference was found between rPocMSP4 and PowMSP1 to anti-rPocMSP4 sera (*p *= 0.9063) (**c**) and between rPowMSP4 and PowMSP1 to anti-rPowMSP4 sera (*p *< 0.0430) (**d**)

The specificity and reactivity of antibodies raised against PoMSP4 proteins were evaluated by testing rPoAMA1 or rPoMSP1 proteins with mouse anti-rPoMSP4 sera from each immunized group using ELISA. All sera from mice immunized with rPoMSP4 recognized the antigen when the ELISA plate was sensitized with rPoAMA1 proteins (Figs. 5a and 5b), and no significant difference in cross-reactivity was observed (*p *> 0.05). However, significant differences in cross-reaction were detected between rPocMSP1 to anti-rPocMSP4 sera and rPocMSP4 to anti-rPocMSP4 sera (*p *< 0.0001); whereas no significant difference was observed between rPocMSP4 and PowMSP1 to anti-rPocMSP4 sera (*p *= 0.9063; Fig. 5c). In addition, cross-reactivity was observed between rPocMSP1 to anti-rPowMSP4 sera and rPowMSP4 to anti-rPowMSP4 sera (*p *< 0.0001), as well as between rPowMSP4 and PowMSP1 to anti-rPowMSP4 sera (*p *< 0.0430; Fig. 5d). In summary, anti-rPoMSP4 sera cross-reacted against rPocAMA1 or rPowAMA1, and rPowMSP1 proteins; by contrast, they showed low reactivity with rPocMSP1 proteins. This result suggested that rPoMSP4 antigens were able to generate antibodies with broad immune responses against such important proteins (known to be associated with red blood cell invasion [15]), making them attractive candidates to induce a polyclonal immune response.

## Discussion

The effectiveness of malaria vaccines mostly depends on the high degree of conservation and excellent level of immunogenicity. The present study analysed 46 *P. ovale* clinical isolates imported from Africa. Amplification and sequencing results showed that the full lengths of *pomsp4* nucleotide and PoMSP4 amino acid sequences (from 23 *P. o. curtisi* isolates and 23 *P. o. wallikeri* isolates) were completely conserved. These results were in accordance with other reports that showed a high degree of conservation of *msp4* orthologs in *P. falciparum* isolates [33, 34, 37]. In addition, the high degree of conservation suggested that *pomsp4* can be useful in PCR-based diagnostic testing for *P. ovale* spp.

The development of blood-stage vaccines against malaria focuses on surface-exposed proteins of erythrocytes infected with the malaria parasites, which are accessible for antibodies. Thus, humoral immune responses are important against malaria parasites because they play essential roles in protecting against blood-stage malaria [38, 39]. Antibody responses against blood-stage antigens are known for their importance in protecting against malaria [40], and this study showed that recombinant PoMSP4 proteins were immunogenic in mice. IgG antibody titres elicited by PocMSP4 and PowMSP4 fragments were substantially higher than those found in the control group (PBS), and sera from immunized mice showed positive reactivity with rPocMSP4 and rPowMSP4 proteins. Cross-reaction between rPocMSP4 and rPowMSP4 was detected through ELISA and Western blot analyses. These results suggested that rPoMSP4 shared similar antigenic determinants that could enable the measurement of species-specific efficacy in vaccine trials. Such high cross-reaction between the two proteins addressed the possibility that both proteins may be used together for vaccine design. However, this result would need future functional studies to address the possibility of vaccine development. Antibody avidity seems to play an important role in protective immunity [41]. Results of this study indicated that rPocMSP4 and rPowMSP4 induced high-avidity antibodies (PocMSP4: 80.50% and PowMSP4: 92.32%). These findings suggested that MSP4 is a valid model for understanding the immune response in mice. MSP1 and AMA1 proteins are currently being developed as subunits for the development of malaria vaccines [19, 42, 43]. In the present study, cross-antisera reaction between PoMSP4 and PoAMA1 or PoMSP1 was observed, that is, anti-PoMSP4 antiserum cross-reacted with recombinant PoAMA1 and PoMSP1 in ELISA. This ability of PoMSP4 antigens to generate antibodies with broad immune responses against such important proteins make them attractive candidates to induce a polyclonal immune response.

Lymphocytes play an important role in the immune system because these cells determine the specificity of the immune response to infectious microorganisms and other foreign substances. Lymphocyte proliferation assays are widely used to measure cell-mediated T-cell immunity and responses to specific antigens [44, 45]. Besides humoral responses, the cellular immune response is also an important indicator for evaluating the immunogenicity of a vaccine candidate. As demonstrated by the results, of this study PocMSP4 and PowMSP4 proteins could induce cellular immune responses in mice, suggesting that humoral and cellular immune responses play crucial roles for PoMSP4 in protection.

## Conclusion

Immunization with conserved rPoMSP4 protein fragments resulted in a remarkable humoral immune response. Cross-reaction between rPocMSP4 and rPowMSP4 proteins or between rPoAMA1 and rPoMSP1 proteins to anti-rPoMSP4 was detected, suggesting that these proteins shared similar antigenic determinants. PoMSP4 could induce cellular immune responses. Though future studies are needed to assess the mechanism by which PoMSP4 fragments play roles in immune protection, but the fragments of *P. ovale* that were used in this study may be more widely evaluated as potential vaccine candidates.

## Supplementary information

**Additional file 1: Table S1.** Information on imported *Plasmodium ovale curtisi* and *Plasmodium ovale wallikeri* used in this study.

**Additional file 2: Table S2.** The *msp4* Gene ID number of other *Plasmodium* species.

**Additional file 3: Figure S1.** Alignments for the amino acid sequences of *Plasmodium ovale curtisi* and *Plasmodium ovale wallikeri* MSP4 in all amplified clinical isolates **a.** PocMSP4 amino acid sequences **b.** PowMSP4 amino acid sequences.

## Data Availability

The data supporting the conclusions of this article are included within the article and its additional files.

## Abbreviations

MSP4: Merozoite surface protein4
rPoMSP4: Recombinant PoMSP4
PCR: Polymerase chain reaction
ELISA: Enzyme-linked immunosorbent assays
