# Supplementary material for: Immunogenicity analysis of conserved fragments in Plasmodium ovale species merozoite surface protein 4
Source: Malar J. 2020 Mar 30;19:126. doi: 10.1186/s12936-020-03207-7 (PMC7106901; doi:10.1186/s12936-020-03207-7)
Supplement: Supplementary file 1 — Additional file 1: Table S1. Information on imported Plasmodium ovale curtisi and Plasmodium ovale wallikeri used in this study. [file 12936_2020_3207_MOESM1_ESM.docx]

**Information on imported P. ovale curtisi and P. ovale wallikeri clinical isolates**

| Species confirmation | Isolate number | Gender | Country of Origin | Parasitaemia level |
| --- | --- | --- | --- | --- |
| *P. ovale curtisi* | Poc-1  Poc-14  Poc-17  Poc-22  Poc-27  Poc-34  Poc-46  Poc-50  Poc-57  Poc-58  Poc-59  Poc-60  Poc-62  Poc-64  Poc-65  Poc-67  Poc-68  Poc-69  Poc-70  Poc-71  Poc-81  Poc-86  Poc-87 | Male  Male  Male  Male  Male  Male  Male  Male  Male  Male  Male  Male  Male  Male  Male  Male  Male  Male  Male  Male  Male  Male  Male | Angola  Angola  Equatorial Guinea  Equatorial Guinea  Equatorial Guinea  Equatorial Guinea  Republic of Congo  Republic of Congo  Congo-Kinshasa  Guinea  Ghana  Gabon  Cameroon  Cameroon  Cameroon  Liberia  Liberia  Mozambique  South Africa  Niger  Nigeria  Zambia  Zambia | 9377  5922  4391  7663  8834  11025  16753  2126  2021  2200  4053  9363  2736  3800  3239  4500  5381  25460  5532  2500  3651  5424  2987 |
| *P. ovale wallikeri* | Pow-3  Pow-7  Pow-8  Pow-13  Pow-14  Pow-17  Pow-20  Pow-21  Pow-22  Pow-23  Pow-25  Pow-30  Pow-50  Pow-51  Pow-56  Pow-59  Pow-67  Pow-73  Pow-76  Pow-77  Pow-80  Pow-84  Pow-90 | Male  Male  Male  Male  Male  Male  Male  Male  Male  Male  Male  Male  Male  Male  Male  Male  Male  Male  Male  Male  Male  Male  Male | Angola  Angola  Angola  Angola  Angola  Angola  Angola  Angola  Angola  Equatorial Guinea  Equatorial Guinea  Equatorial Guinea  Equatorial Guinea  Equatorial Guinea  Republic of Congo  Republic of Congo  Gabo  Cote d’ Ivoire  Mozambique  Mozambique  Nigeria  Nigeria  Uganda | 10971  34793  20029  2172  1837  12435  5615  3619  8597  9339  1829  4168  2573  12502  208000  1800  10370  4850  2111  13000  3613  3135  2290 |
