# Supplementary material for: Immunogenicity analysis of conserved fragments in Plasmodium ovale species merozoite surface protein 4
Source: Malar J. 2020 Mar 30;19:126. doi: 10.1186/s12936-020-03207-7 (PMC7106901; doi:10.1186/s12936-020-03207-7)
Supplement: Supplementary file 2 — Additional file 2: Table S2. The msp4 Gene ID number of other Plasmodium species. [file 12936_2020_3207_MOESM2_ESM.docx]

**Table S2** The *msp4* Gene ID number of other *Plasmodium* species

| Species | Strain | Gene ID from PlasmoDB |
| --- | --- | --- |
| *P. falciparum* | KE01 | PfKE01_020009500 |
|  | TG01 | PfTG01_020012000 |
|  | IT | PfIT_020011700 |
|  | HB3 | PfHB3_020011700 |
|  | GB4 | PfGB4_020010000 |
|  | CD01 | PfCD01_020011900 |
|  | 3D7 | PF3D7_0207000 |
|  | Dd2 | PfDd2_020009800 |
|  | ML01 | PfML01_020009900 |
|  | KH02 | PfKH02_020011000 |
|  | 7G8 | Pf7G8_020011700 |
|  | SD01 | PfSD01_020012500 |
|  | GA01 | PfGA01_020010000 |
|  | GN01 | PfGN01_020012300 |
|  | KH01 | PfKH01_020012200 |
|  | SN01 | PfSN01_020010000 |
| *P. reichenowi* | CDC | PRCDC_0206000 |
| *P. ovale curtisi* | GH01 | PocGH01_04023000 |
| *P. ovale wallikeri* | PowCR01 | PowCR01_040018600 |
| *P. gallinaceum* | 8A | PGAL8A_00017500 |
| *P. relictum* | SGS1-like | PRELSG_0415100 |
| *P. malariae* | UG01 | PmUG01_04025600 |
| *P. knowlesi* | strain H | PKNH_0414100 |
| *P. vivax* | P01 | PVP01_0418300 |
|  | Sal-1 | PVX_003775 |
| *P. cynomolgi*  *P. billicollinsi* | strain M  G01 | PcyM_0417800  PBILCG01_0209600 |
