# Supplementary figures and images for: Immunogenicity analysis of conserved fragments in Plasmodium ovale species merozoite surface protein 4
Source: Malar J. 2020 Mar 30;19:126. doi: 10.1186/s12936-020-03207-7 (PMC7106901; doi:10.1186/s12936-020-03207-7)

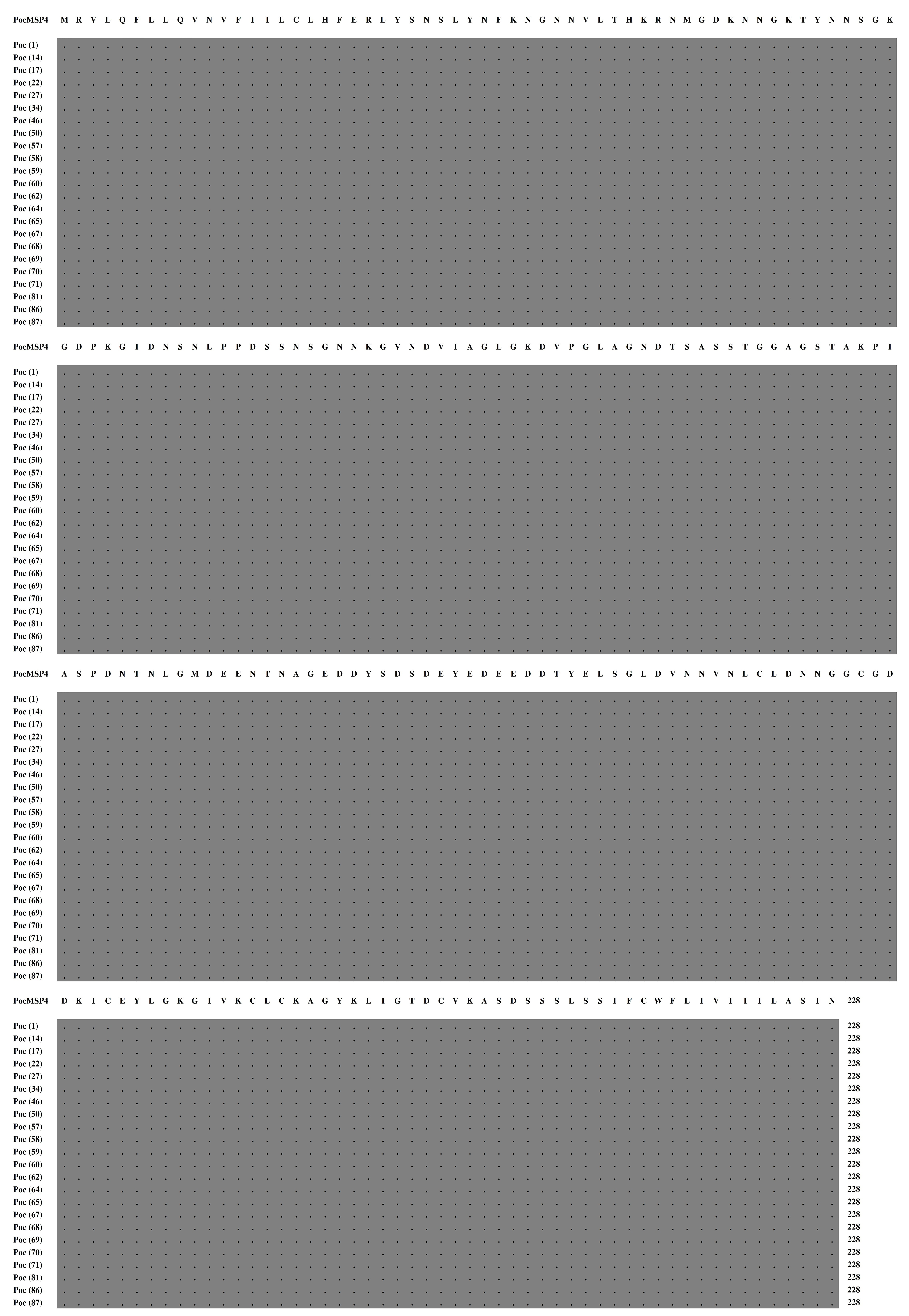

Supplement: Supplementary file 3 — Additional file 3: Figure S1. Alignments for the amino acid sequences of Plasmodium ovale curtisi and Plasmodium ovale wallikeri MSP4 in all amplified clinical isolates a. PocMSP4 amino acid sequences b. PowMSP4 amino acid sequences. [file 12936_2020_3207_MOESM3_ESM.tif]
